# A paper and plastic device for the combined isothermal amplification and lateral flow detection of *Plasmodium* DNA

**DOI:** 10.1186/s12936-015-0995-6

**Published:** 2015-11-26

**Authors:** Michael S. Cordray, Rebecca R. Richards-Kortum

**Affiliations:** Rice University Department of Bioengineering, 6100 Main St., Houston, TX 77005 USA

**Keywords:** Paper microfluidics, Recombinase polymerase amplification, Nucleic acid test

## Abstract

**Background:**

Isothermal amplification techniques are emerging as a promising method for malaria diagnosis since they are capable of detecting extremely low concentrations of parasite target while mitigating the need for infrastructure and training required by other nucleic acid based tests. Recombinase polymerase amplification (RPA) is promising for further development since it operates in a short time frame (<30 min) and produces a product that can be visually detected on a lateral flow dipstick. A self-sealing paper and plastic system that performs both the amplification and detection of a malaria DNA sequence is presented.

**Methods:**

Primers were designed using the NCBI nBLAST tools and screened using gel electrophoresis. Paper and plastic devices were prototyped using commercial design software and parts were cut using a laser cutter and assembled by hand. Synthetic copies of the *Plasmodium* 18S gene were spiked into solution and used as targets for the RPA reaction. To test the performance of the device the same samples spiked with synthetic target were run in parallel both in the paper and plastic devices and using conventional bench top methods.

**Results:**

Novel RPA primers were developed that bind to sequences present in the four species of *Plasmodium* which infect humans. The paper and plastic devices were found to be capable of detecting as few as 5 copies/µL of synthetic *Plasmodium* DNA (50 copies total), comparable to the same reaction run on the bench top. The devices produce visual results in an hour, cost approximately $1, and are self-contained once the device is sealed.

**Conclusions:**

The device was capable of carrying out the RPA reaction and detecting meaningful amounts of synthetic *Plasmodium* DNA in a self-sealing and self-contained device. This device may be a step towards making nucleic acid tests more accessible for malaria detection.

## Background

There is growing interest in developing nucleic acid tests (NATs) that can be used to diagnose and monitor infectious diseases in low-resource areas [1, 2]. NATs are extremely sensitive and highly specific, but their widespread use at the point-of-care is hindered by high per-test cost, technical complexity and infrastructure requirements [3–5]. Isothermal amplification methods offer a promising avenue to reduce the cost and complexity of NATs, since they do not require a thermocycler, thereby increasing global access to high quality diagnostics [6–9].

Recombinase polymerase amplification (RPA) is a recently developed isothermal amplification method that can produce detectable results in less time and at lower temperatures than other isothermal amplification techniques; these advantages make it particularly suitable for use at the point-of-care. The RPA reactions operates in a manner similar to conventional PCR, using a forward and reverse primer to amplify a sequence of DNA. However instead of using heat to denature the target strand, the RPA reaction operates isothermally by using enzymes. Recombinase is added to the master mix, which forms complexes with the primer strands. These recombinase-primer complexes then displace the anti-sense strand of the target, binding the primers to the target strands and creating binding sites for polymerase to bind [10]. The RPA reaction can be modified (RPA nfo) with an internal probe sequence and a labelled primer to generate a product which can be detected by a commercial lateral flow dipstick assay, allowing for visual readout of the result [11, 12].

Paper microfluidic devices offer a promising platform to translate isothermal amplification tests to the point-of-care, since they are made of inexpensive materials, do not require pumps or other external apparatus and can replicate many of the functions of more traditional microfluidic devices [13, 14]. Recently, Rohrman et al. presented a foldable paper and plastic microfluidic device to carry out the RPA reaction [15]. This system was used to amplify a target sequence in the HIV gag gene, and achieved detectable amplification of as few as 10 copies of HIV DNA in as few as 15 min. However a key limitation of this system was that it required the user to cut the device open to extract and dilute the amplified product for detection on lateral flow strips. Without dilution, the crowding reagent used in the RPA nfo buffer leads to false positive results on the lateral flow detection strips. The need to cut open the device and dilute the reaction products adds additional user steps and greatly increases the likelihood of contamination of the workspace with the amplified target, which in turn can lead to false positive results in future tests.

Here, an integrated device capable of carrying out isothermal amplification using the RPA reaction, post-amplification dilution, and lateral flow detection of the resulting product is presented. This device is self-sealing and once loaded and sealed contains all reagents and mechanisms necessary to amplify, dilute and detect the product without the need to open the system.

As an example of a disease which would benefit from increased access to NAT-based diagnostics, this integrated system was used to detect synthetic copies of a gene present in *Plasmodium*, the parasite which causes malaria [16]. There is a need for increased access to point-of-care malaria diagnostics which can detect the low-level infections which are often missed by thin and thick smear microscopy, the current gold standard [17–19]. Several isothermal amplification techniques have been investigated as a method for diagnosing malaria, but RPA nfo offers advantages due to its short run time and easy visual detection scheme [20–27]. RPA nfo primers and a probe were developed which target a region common to all human infectious species of *Plasmodium*. The integrated paper and plastic system can be used to detect clinically relevant amounts of a synthetic copy of the *Plasmodium* 18S gene.

## Methods

### Materials

RPA nfo amplification kits and lateral flow detection strips (Milenia Hybridtech 1) were obtained from TwistDx Limited (Cambridge, UK). DNA primer, probe and target plasmid sequences were obtained from Integrated DNA Technologies (Coralville, IA, USA) and all except the plasmid prepared by suspending them in water to a concentration of 10 µM. Materials for plasmid cloning and purification were ordered from Qiagen Inc. (Valencia, CA, USA). Sheets of 0.003″ acetate (KD3CL0811) and double-stick tape (KDT912-12) were obtained from Grafix (Maple Heights, OH, USA). Blotter paper (CFSP223000) and glass fiber (GFCP203000) pads were obtained from Millipore Corp (Billerica, MA, USA). Whatman 1 Chromatography paper was obtained from Fisher Scientific Company LLC (Pittsburgh, PA, USA). Agarose, TAE, ethidium bromide, TBST and water were obtained from Sigma Aldrich (St. Louis, MO, USA). Screw top microcentrifuge tubes (used for all bench top reactions) were obtained from Genesee Scientific Corp (San Diego, CA, USA). 25 g metal weights were obtained from Amazon (Seattle, WA, USA).

### Primer design and screen

Primers and a probe specific to the *Plasmodium* 18S gene were designed based on guidelines provided by TwistDx, the manufacturer of the RPA reaction, and were modified with end-tags and internal modifications to make them compatible with lateral flow detection. The target selected is the 18S gene because it is highly conserved, and contains regions shared amongst all species of *Plasmodium* which commonly infect humans [28, 29]. For testing purposes, the target used was a synthetic plasmid containing a 2090 bp copy of the malaria 18S gene (total size of plasmid 5091 bp) (GenBank: MS19172). This plasmid was cloned into *E. coli* to increase copy number and purified using standard protocols [30]. Successful cloning and purification of the gene was confirmed by gel electrophoresis on a 3 % agarose gel stained by ethidium bromide. For all experiments, this purified target was then diluted in water to a concentration of 200, 50 and 5 copies/µL before being input to RPA nfo reactions along with an additional no target control of water.

The NCBI’s nucleotide BLAST tools were used to search for primers specific to *Plasmodium* without significant overlap with other genomes. A variety of forward and reverse primer candidates were chosen and pairs were screened by observing their performance on a 3 % agarose gel stained with ethidium bromide and imaged on a Bio-Rad Gel Doc XR+. Once the outer primer pairs were chosen, internal probes were screened through a similar process. The set of primers and probe with the brightest band on the gel were selected for further experimentation:

- Forward primer: 5′-CACGAACTAAAAACGGCCATGCATCACCATCC-3′
- Reverse primer: 5′-biotin-CCTTATGAGAAATCAAAGTCTTTGGGTTCTGGGG-3′
- Probe: 5′-FAM-ATCAAGAAAGAGCTATTAATCTGTCAATCCTAC-THF-CTTGTCTTAAACTAGTG-SpC3-3′

The selected primers and probe target a sequence present in the genome of all major species of *Plasmodium,* which infect humans, based on BLAST searches of the NCBI nucleotide database. This set of primers and probe produce a 218 bp primary product labelled only with biotin, as well as a 181 bp secondary product which is labelled for detection on the lateral flow strips.

To generate a product detectable on lateral flow strips, the 5′ end of the reverse primer is labelled with a biotin. A probe was designed that is labeled on its 5′ end with a FAM group, a C3 spacer (SpC3) on the 3′ end and a tetrahydrofuran residue (THF) which replaces an internal base. The RPA reaction produces a primary product which is labelled with a biotin tag from the reverse primer. The probe binds internally to this primary product, and once bound, the nfo enzyme in the reaction mix removes the THF and the bases 3′ to it, including the C3 spacer. This allows the remaining bases of the probe to act as a new forward primer that produces a secondary product labelled with both biotin and FAM. This secondary product was detected using lateral flow strips functionalized with streptavidin to capture the product at the test line and gold nanoparticles functionalized with anti-FAM to produce a color change at the test line in the presence of the labelled secondary product.

### RPA reaction in solution

The RPA nfo reaction was carried out following the manufacturer’s recommended protocols [10]. In brief, a master mix was created containing 2.1 µL each of 10 µM of each primer, 0.6 µL of 10 µM of the probe, 3.2 µL of water and 29.5 µL of the reaction buffer included with the kit. In all experiments 37.5 µL of master mix was aliquoted into individual reaction tubes containing the freeze-dried reaction pellet included with the kit. To each of these tubes 10 µL of target was added, and then 2.5 µL of 280 mM magnesium acetate (MgAc) was added to the cap of the reaction tube. Tubes were then briefly centrifuged and vortexed to mix MgAc with the other reagents. The reaction tubes were then incubated for 30 min in a heat block at 37 °C.

### Lateral flow detection and imaging

Immediately after incubation, RPA reactions were removed from heat and 2 µL was removed and added to 98 µL of tris-buffered saline with 0.05 % tween-20 (TBST), which was used as both sample diluent and lateral flow running buffer. This dilution step is necessary because the dextran sulfate used as a crowding reagent in the RPA nfo buffer causes non-specific binding of the antibody labelled gold to the test line, generating a false positive. After dilution, 10 µL of the product was removed and spotted onto the end of a lateral flow strip. The strip was placed in a well containing 98 µL of TBST and allowed to run for 5 min. The strips were then scanned using a commercial document scanner (Epson Perfection V500 Photo) and images were recorded at 1200 dpi using the ‘reflective document’ settings.

The signal-to-background ratio (SBR) of the lateral flow images were analysed using a custom MATLAB script. The user manually segments the test line and the surrounding background region. The SBR is calculated by calculating the ratio of the average intensity of the signal and background regions [31].

### RPA reaction in paper and plastic device

#### Device description

**Fig. 1:**
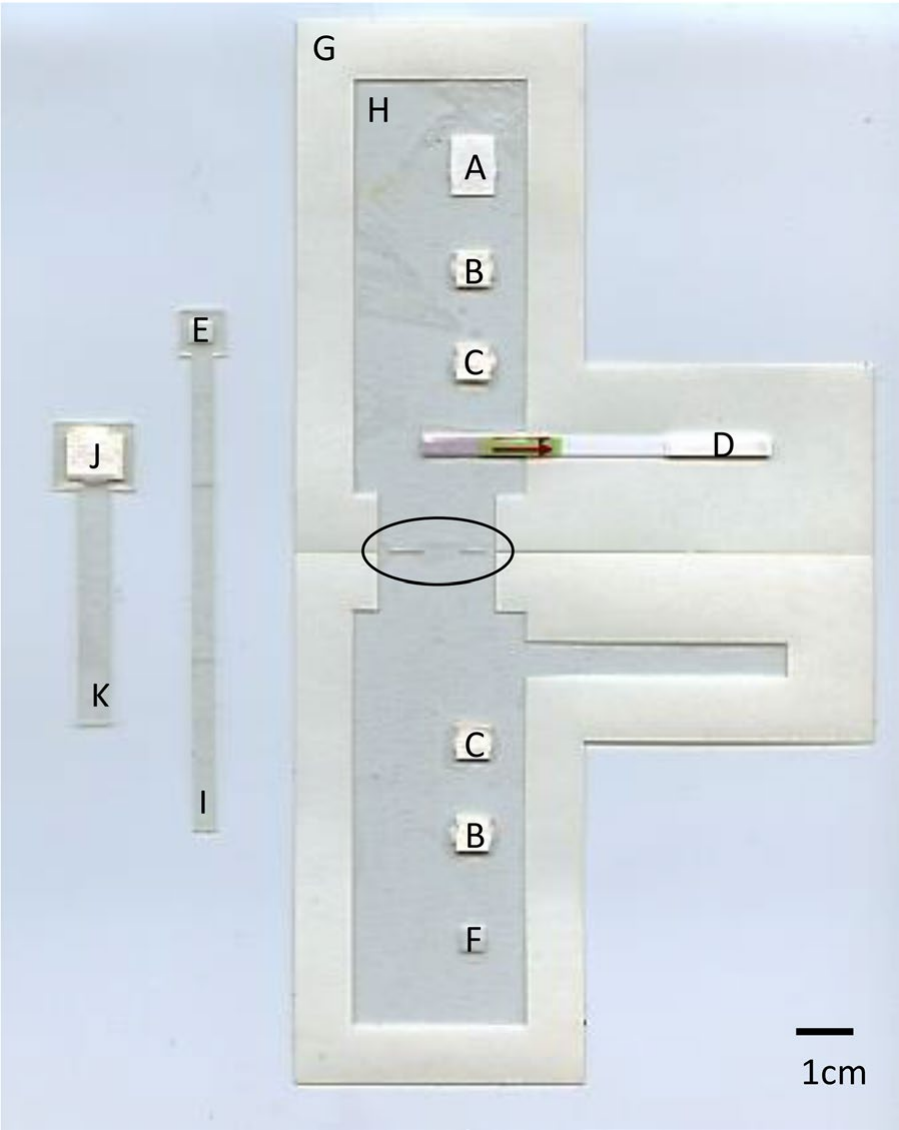
Layout and components of the paper and plastic amplification and detection device. The device is shown in its configuration just before it would be loaded with wet reagents. The sample sliders are shown off to the side for clarity. The slots where they would be inserted, which are cut into the hinge where the device folds shut, are *highlighted* with an *oval outline*. The components of the device are *A* the RPA pad; *B* the dry dilution pads; *C* the wet dilution pads; *D* the lateral flow strip; *E* the sample pad; *F* the mixing pad; *G* the base layer; *H* the acetate mask; *I* the sample slider; *J* the running buffer pad; and *K* the running buffer slider

The paper and plastic device needs to carry out three main functions: (1) amplify the target using RPA; (2) dilute the resulting product; and (3) detect the product using a lateral flow sandwich assay. In addition, the device must transfer the product between the amplification, dilution, and detection modules. A sequence of paper pads loaded with various reagents was used to carry out these functions. The amplification reaction is carried out on the RPA pad (Fig. 1A): a rectangular piece of Whatman number 1 paper which holds the RPA nfo reagents. The dilution function is carried out by four dilution pads (small squares of blotter paper), in two separate stages of dilution (Fig. 1B, C). Detection is carried out using a commercial lateral flow strip (Fig. 1D) with running buffer delivered via a running buffer pad. To move amplified product from the amplification zone to each of the two dilution zones and then to the lateral flow strip, the sample pad (Fig. 1E), is physically moved across the other parts of the device. When the sample pad is placed in contact with another pad, passive diffusion results in target transport to a subsequent zone of the device. To improve mixing during the amplification step, an extra pad of glass fiber is placed so that it will be in contact with the sample pad when the sample pad is in contact with the RPA pad. This mixing pad (Fig. 1F) helps promote the diffusion of the RPA master mix onto the sample pad.

The case of the device is made of a symmetric L-shaped piece of acetate backed double-stick tape (Fig. 1G). The RPA pad, dilution pads and lateral flow strip are all attached to this base layer. The RPA pad is placed at the top of the L, followed by pairs of dilution pads along the vertical section of the L, and the lateral flow strip is placed along the base of the L. An acetate mask (Fig. 1H) is adhered to the remaining exposed adhesive of the base layer to prevent the device from adhering to itself when folded shut for use.

A slider is used to move the sample pad through the system. The slider holds the sample pad between two layers of acetate-backed tape with a hole cut through it to expose the surface of the sample pad. The slider has a long tail which sticks out through a slot in the base layer and is pulled to slide the sample pad between the different functional parts of the device (Fig. 1I). A second slider holds a running buffer pad (running buffer pad Fig. 1J, running buffer slider Fig. 1K), which is used to deliver running buffer to the lateral flow strip.

#### Device assembly

All device components were cut using a 60 W CO_2_ laser cutter (Universal Laser Systems, Scottsdale, AZ, USA). One side of a double stick tape sheet was exposed and adhered to acetate prior to cutting. Acetate backed pieces of tape were cut with contact paper side up. The following settings were used for each material: acetate 3 % power, 10 % speed; blotter paper 5 % power, 5 % speed; Whatman 1 paper 3 % power, 5 % speed; glass fiber 3 % power, 5 % speed; and cuts through the double-stick tape 7.5 % power, 10 % speed. After the acetate and tape base layer was cut, the outlines for each component were scored using a setting that cuts only through the contact paper (1.8 % power, 12 % speed).

To assemble the device, the contact paper was removed from the scored areas and the four dilution pads and the lateral flow strip were adhered to the base layer. The RPA nfo reaction pellet was placed on the exposed tape and the RPA pad pressed down on top to crush the pellet. The remaining paper (except for the separate border piece) was removed and the acetate mask was adhered to the base. The sample and running buffer sliders were assembled. The tails of the finished sliders were threaded into the slots cut in the base layer.

#### Operation of device

To operate the device, the remaining border of contact paper is removed to expose the sticky edge that will be used to seal the device closed. The device is then loaded with wet reagents necessary for amplification, dilution and detection. 100 µL of TBST is added to the running buffer pad. An additional 40 µL of TBST is added to the two wet dilution pads. 37.5 µL of the RPA nfo master mix is added to the pad covering the RPA nfo pellet. Finally 10 µL of the sample is added to the sample slider, along with 2.5 µL of MgAc. The device is then folded in half and sealed shut.

**Fig. 2:**
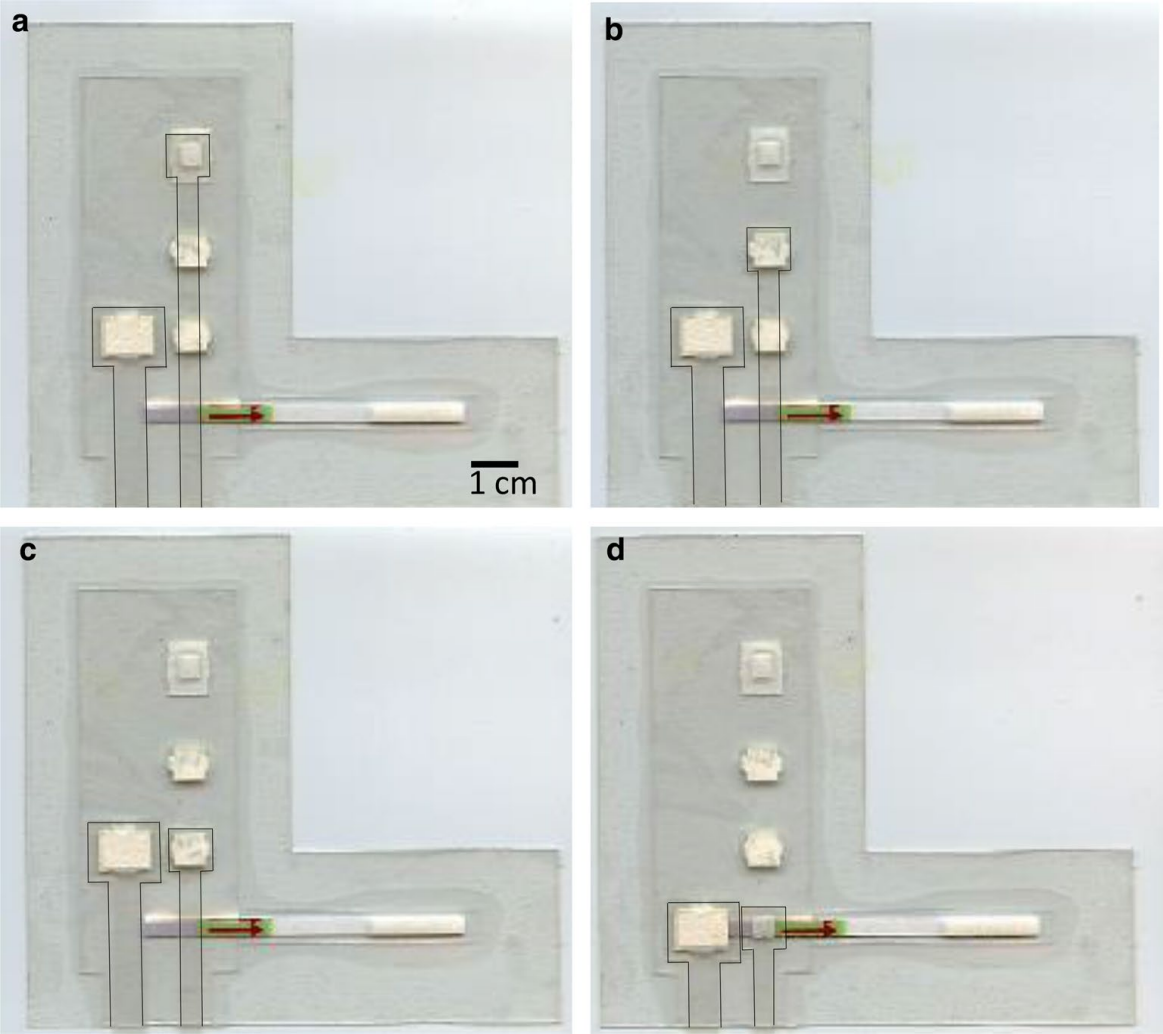
Device in operation. For clarity the weight which would rest over the location of the sample pad has been omitted and the edges of the sample and running buffer sliders have been outlined. **a** Sample slider at position 1, mixing RPA reagents and target and allowing amplification to occur (30 min). **b** After amplification, the sample slider pulled down to position 2, in contact with dry dilution pads to absorb RPA buffer (10 min). **c** The sample slider pulled down to position 3, in contact with wet dilution pads, for dilution with TBST (10 min). **d** Sample slider pulled down to position 4 into contact with lateral flow strip. The running buffer slider is pulled down to activate the strip (5 min)

The sample slider begins centered over the RPA pad, position 1, so that when the device is sealed shut, the sample and RPA reagents mix together. To begin amplification, the entire device is placed on a hot plate set to 37 °C and a 25 g metal weight is placed over the sample slider to ensure even heating and consistent mixing of reagents (Fig. 2a). After 30 min, the device is removed from the hot plate and the sample slider is pulled down to position 2, placing it in contact with the dry dilution pads and the weight is placed over top of it (Fig. 2b). After 10 min, the slider and weight are moved to position 3 in contact with the wet dilution pads for ten more minutes (Fig. 2c). Finally, the sample pad is moved down to position 4 over top of the lateral flow strip, the running buffer slider is also pulled down to the strip, and the weight is placed on top (Fig. 2d). The strip is allowed to run for 5 min, and then the device is scanned.

To test and optimize the dilution zone, 0.25 % Ponceau S dye was used in place of sample to monitor the movement of fluid through the system. All other components were loaded as if an amplification reaction was to be run. After amplification and dilution, rather than running the lateral flow strip, the device was cut open and the sample pad was removed and placed in 90 µL of TBST, vortexed, and the concentration of dye in solution was measured using a Cary 50 Spectrophotometer.

## Results

**Fig. 3:**
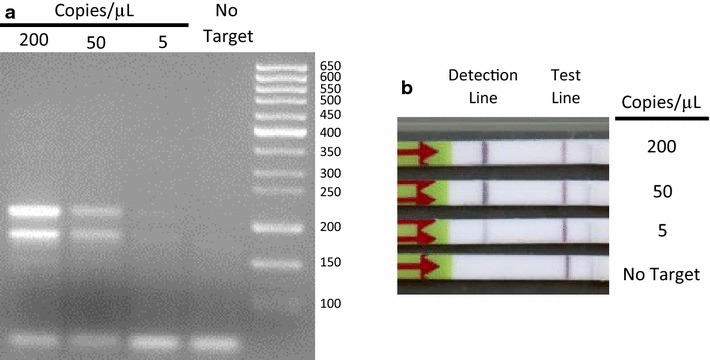
Performance of RPA nfo reaction and *Plasmodium* primers and probes in benchtop reactions. **a** An agarose gel stained with ethidium bromide showing the amplification of the primary (218 bp) and secondary (181 bp) products. **b** An example of the products run on lateral flow strips. Note that the positive result at 5 copies/µL of target is easier to determine on the strips than on the gel

The *Plasmodium* primers produced the expected primary and secondary amplicons based on agarose gel analysis of the products (Fig. 3a) and achieved a limit of detection (LOD) of 5 copies/µL (50 total copies) using visual inspection of the lateral flow strips (Fig. 3b). The visual result was confirmed by using SBR to objectively determine if a strip was positive or not. A strip was considered positive if it had a SBR that was greater than three standard deviations above the averaged SBR of negative control strips. Using this cutoff, the limit of detection of the assay, at which all samples were positive in every test, was also 5 copies/µL (50 total copies).

Using dye to track the flow of fluids, the slider system was capable of reliably moving fluids through the system and transferring them to subsequent pads. The number of dilution pads, their size, the amount of buffer they were loaded with and the time spent on each pad were all varied as experimental conditions. After optimization of the dilution system, two pairs of blotter paper pads were used, one wet and one dry, to achieve the necessary dilution. The dry dilution pads were used to remove some fluid on the sample pad so that it could be replaced with TBST at the wet dilution pads. It was found that by letting the fluids exchange for 10 min on each set of pads under a 25 g weight, a final dilution of 1:49 (2.04 % ± 0.26 % dilution, n = 5) could be achieved. No false positives due to lack of RPA buffer dilution were observed using this design.

**Fig. 4:**
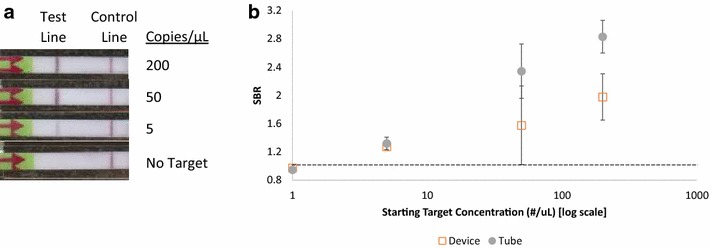
Demonstration of performance of paper and plastic device. **a** Representative images of the lateral flow strips run in the devices. **b** Comparison of the averaged SBR of samples run in the device and in tubes (n = 3), the *error bars* represent ±1 standard deviation. Strips were positive by SBR if they were more than 3 standard deviations above the averaged no target control SBR, shown as a *dotted line* on the figure

The total run time for the device is 55 min (30 min incubation, 20 min dilution and 5 min lateral flow detection). Including the loading and operation of the device, the entire assay can be carried out in about an hour. To test the performance of the system, the same samples were simultaneously run side-by-side using both conventional bench top methods and the paper and plastic devices. Both methods were found to have a limit of detection of 5 copies/µL (50 total copies) using visual inspection and objective analysis by SBR (Fig. 4). Although the limit of detection was unaffected, the devices consistently had a lower SBR than the assay run on the bench top. At both 200 and 50 copies/µL there was approximately a 30 % reduction in the average SBR. There was less than a 5 % difference at both 5 copies/µL and in the no target controls. The reduced SBR at higher target concentrations may be due to two factors: running the assay in a paper matrix may reduce the mixing in the system compared to running it in a tube. Additionally, it was found that the plastic layered overtop of the lateral flow strip increases the signal over both the test line and the background of the strip driving the SBR closer to unity.

## Discussion

A novel set of primers have been developed which detects a sequence shared amongst the major human infectious species of *Plasmodium* and which can detect at least 5 copies/µL (50 total copies) of a plasmid containing a synthetic copy of a *Plasmodium* gene. This detectable concentration is comparable to other isothermal amplification methods for malaria and below the LOD of thin smear microscopy, the gold standard for malaria diagnosis [16, 32]. These primers are used with RPA nfo, an isothermal amplification method which can run at relatively low temperatures, which produces results in as few as 35 min when run on the bench top. A commercially available assay for malaria which uses the LAMP isothermal amplification technique has been developed which is capable of detecting around 2 copies/µL (5 total copies) [26, 27]. Although the RPA nfo reaction detects fewer copies, the concentration at which it becomes positive is on the same order as LAMP and other isothermal techniques, and RPA nfo may offer advantages such as a shorter amplification run time and a readout on lateral flow strips [16].

A paper and plastic device was created which is capable of running the amplification reaction, diluting the amplified product by a repeatable amount and running the results on an included lateral flow dipstick. The device is made of simple components, can be assembled by the user and uses a novel slider method to transport reagents through the system. The equipment needed beyond the device itself and the sample to be tested are a hot plate capable of 37 °C, a reusable 25 g metal weight, the RPA master mix, the running/dilution buffer (TBST) and pipettes to load these reagents onto the device dilution, running buffer, RPA and sample pads. This device runs the entire assay, including detection, in around an hour and has a limit of detection equivalent to when the assay is run using conventional methods on the bench top. Excluding the cost of the lateral flow strip and the RPA nfo reaction pellet, the device costs around $1.05. The commercial lateral flow strips cost around $3.14 each, and the primers and RPA nfo reaction kits together cost approximately $4.09. This gives an approximate total reaction cost of $8.28 per test, most of which is due to the cost of the commercial components rather than the paper and plastic device.

A key limitation of the current work is that while a self-contained system for running the reaction was presented, it requires the user to add set amounts of fluid to various pads before sealing and operating the device. This means that the system requires roughly the same amount of manipulation as running the reaction on the bench top. Future work could reduce the external parts needed by integrating buffer and primer storage into the device, perhaps by drying them on the pads. In addition, the dilution system used increases the runtime of the assay by 20 min. Although much of this time is hands-off, future work should increase the run speed of the device by developing a more efficient method for diluting the sample. A study should be also be conducted to ensure that the seal of the device is adequate, and that aerosols cannot escape from the device in sufficient quantity to become a source of future contamination.

This study was aimed at developing a proof-of-concept device and used synthetic targets. In order for the paper and plastic devices to be more useful in real world settings, the assay should be tested and optimized on infected and un-infected blood samples. Clinical samples will offer a number of additional challenges compared to the synthetic targets used in this study such as background human DNA and other blood components. Sample preparation techniques capable of lysing blood and extracting DNA in a manner compatible with the RPA nfo assay will need to be developed. Recently, there have been a variety of studies which have examined methods to run PCR and isothermal amplification on clinical malaria samples with minimal need for extra processing or equipment by using for example, a simple lysis technique such as detergents or heat, adapting the amplification protocol to reduce the inhibitory effects of blood, and/or packaging the lysis and amplification together in a matrix such as a hydrogel [33–35]. Once a suitable sample preparation method has been determined, further work should be done to test the paper and plastic device on clinical samples, and, if possible, to continue to add more functions, such as the sample preparation directly to the device.

## Conclusion

A novel set of primers was developed which amplify a sequence which is common to the human infectious species of *Plasmodium* and operate an isothermal amplification reaction which is rapid and has an easy visual readout. A paper and plastic device was also developed which carries out the amplification of the samples, dilutes the product and runs the result on a lateral flow strip. When tested on synthetic targets, a limit of detection of 5 copies/µL (50 total copies) was found, which matches the performance of the same assay run on the bench top. This device is self-sealing and self-contained once wet reagents are added and needs only a 37 °C heat source, reusable weight and timer. This device is a step towards a fully self-contained and easy to operate low-resource appropriate nucleic acid test for malaria diagnosis.
